# Bizarre leiomyoma of the scrotum: A case report and review of the literature

**DOI:** 10.3892/ol.2014.1930

**Published:** 2014-02-28

**Authors:** ZHENGMING SU, GANHONG LI, YADONG WANG, ZUHU YU, ZEBO CHEN, LIANGCHAO NI, SHANGQI YANG, JIONGXIAN YE, YONGQING LAI

**Affiliations:** 1 Department of Urology, Peking University Shenzhen Hospital, Shenzhen, Guangdong 518036, P.R. China; 2 The Guangdong and Shenzhen Key Laboratory of Male Reproductive Medicine and Genetics, Institute of Urology, Peking University Shenzhen Hospital, Shenzhen PKU-HKUST Medical Center, Shenzhen, Guangdong 518036, P.R. China; 3 Shantou University Medical College, Shantou, Guangdong 515041, P.R. China; 4 Department of Urology, Zunyi Medical College Fifth Affiliated Hospital, Zhuhai, Guangdong 519100, P.R. China

**Keywords:** scrotum, bizarre leiomyoma, tumor

## Abstract

Bizarre leiomyomas of the scrotum are rare benign tumors that are often misdiagnosed. In this study, we present a case of bizarre leiomyoma of the scrotum in a 53-year-old male. The patient presented with a painless scrotal mass that was insidious in the right side of the scrotum with no sudden increase in size. Definitive preoperative diagnosis could not be established; however, following surgical resection of the tumor, a diagnosis of bizarre leiomyoma of the scrotum was determined by pathological examination. The patient was followed up six months following resection and no problems were reported. This is the first reported case of bizarre leiomyoma of the scrotum in China. A supplementary review of previously published cases and literature is also presented.

## Introduction

Leiomyomas are benign tumors that originate from smooth muscle, which have been identified throughout the genitourinary tract (1). Following the uterus, the most common tumor location is the renal capsule. Leiomyomas also occur in the renal pelvis, bladder, spermatic cord, epididymis, prostate and the glans penis (2). Scrotal smooth muscle tumors may be further categorized as leiomyomas, bizarre leiomyomas and leiomyosarcomas. However, bizarre leiomyomas of the scrotum are particularly rare and a PubMed search reveals fewer than 14 reports of symplastic, pleomorphic, bizarre and atypical leiomyomas of the scrotum (3–14) (Table I). Leiomyomas are usually painless in nature; however, they may be associated with pain and the development of hydroceles (3). In contrast to scrotal leiomyosarcomas, scrotal leiomyomas with bizarre nuclei are not hypercellular and they lack mitotic activity (8). As a relatively rare tumor, initial diagnosis and differential diagnosis are complicated, the management of which is usually surgical excision. In the present study, a single case of bizarre leiomyoma of the scrotum is reported, which may be mistaken for other scrotal tumors. This study was approved by the ethics committee of Peking University Shenzhen Hospital (Shenzhen, China) and written informed consent was obtained from the patient.

## Case report

A 53-year-old male presented to his physician with a painless scrotal mass located on the right side, which the patient had first observed 2–3 months previously. The mass had remained stable in size during that period. The patient was admitted to Department of Urology, Peking University Shenzhen Hospital, (Shenzhen, China) for further examination on April 13, 2012, and was determined to be asymptomatic with a normal appetite and no weight changes. The patient did not exhibit any urinary, respiratory, cardiovascular or constitutional symptoms and had not previously undergone surgery. There was no prior history of trauma, inflammation or infection and no significant urological past history. Physical examination revealed the patient was a well-developed and well-nourished male. The patient was afebrile with a heart rate of 92 beats per min, a temperature of 36.5°C, blood pressure of 129/73 mmHg and respiratory rate of 18 breaths per min. The chest was clear to percussion and auscultation, and no masses were palpable on abdominal examination. Physical examination identified a firm, elastic, non-tender mass on the right side of the scrotum, located near the testis. The mass was ~1.0 cm in diameter and no tenderness or erythema was observed. The lesion was not fixed to the skin or adjacent deeper tissue, and no warmth or discharge was noted. Testes on both sides were normal on palpation with no inguinal lymphadenopathy observed.

Laboratory examination revealed that the patient’s hemoglobin concentration was 142 g/l and white blood cell count was 5.84×10^9^/l, with 53.0% granulocytes. Concentrations of glucose, urea nitrogen and serum creatine were 4.87 mmol/l, 9.11 mmol/l and 107.3 μmol/l, respectively. Liver function tests and serum electrolytes were recorded to be within normal limits. The serum levels of certain tumor markers, such as α-fetoprotein and β-human chorionic gonadotropin, were observed to be normal. Following examination by a radiologist, the mass was diagnosed as a sebaceous cyst.

**Figure 1:**
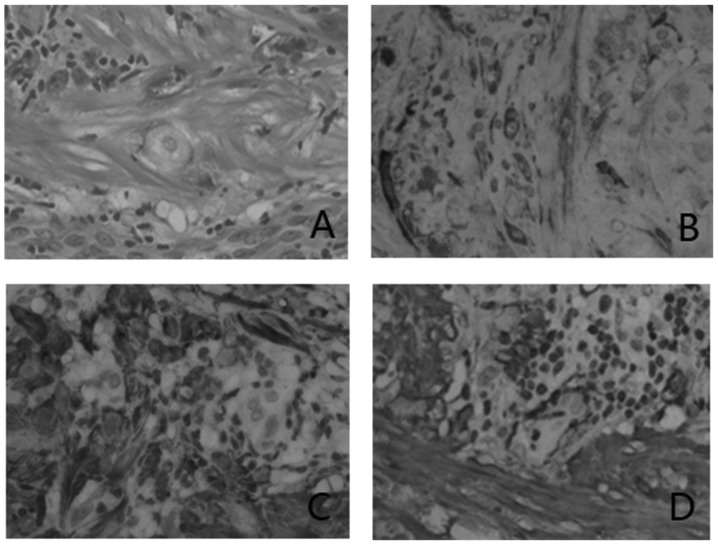
(A) Tumor is composed of interlacing bundles of spindle-shaped muscle cells with bizarre nuclei and occasional nuclear inclusions (hematoxylin and eosin; magnification, ×400). Immunohistochemical staining: Tumor cells demonstrated positive staining for (B) P16, (C) caldesmin and (D) smooth muscle actin (magnification, ×400).

A right percutaneous mass excision was performed on April 17, 2012. The tumor was dissected from the tunica dartos and no invasion of adjacent tissue was observed. The tumor was a solid, well-circumscribed, 1.2×1.0×0.8 cm-sized, oval mass that originated from the tunica dartos, which was independent of the testis, epididymis and funiculus spermaticus. The pathological report revealed clear surgical margins of the tumor. Microscopically, the mass consisted of irregularly shaped cells, with certain tumor cells exhibiting bizarre nuclei and demonstrating focal lymphocytic infiltration into the stroma (Fig. 1). Immunohistochemical staining revealed that the tumor cells were positive for P16, smooth muscle actin and caldesmin (Fig. 1). Following surgical resection of the tumor, the patient was followed up for six months and no problems were reported.

## Discussion

Bizarre leiomyomas of the scrotum are extremely rare; only 14 cases have been reported previously (3–9). In the current study, the fifteenth case of bizarre leiomyoma of the scrotum has been described. This case is the first reported in China. The diagnosis in the present study was mainly based on microscopic analysis and immunophenotype.

Previously reported cases tend to be asymptomatic and painless in nature; therefore, patients may not seek medical consultation for prolonged periods of time, sometimes decades, by which time the tumors may have grown large enough to become cosmetically undesirable or to cause ulceration of the overlying skin. Of the 14 cases, 12 bizarre leiomyomas of the scrotum are solitary, subcutaneous tumors ranged from 0.5 to 10 cm in diameter (Table I). They usually present between the fourth and seventh decades of life and clinically present as a circumscribed swelling or pedunclated scrotal mass. The tumors appear to occur with equal frequency on the right and left side, and are often identified incidentally by physicians during routine physical examinations. Thus far, all of the reported cases of bizarre leiomyoma have been identified as benign tumors and the prognosis has been good.

Ultrasound scans can provide useful information with regard to scrotal mass diagnosis. However, it is difficult to reliably identify malignant scrotal masses on the basis of sonographic features alone. Resection of the mass may be required, as preoperative and intraoperative findings may not be effective in excluding malignancy. However, the frozen section procedure is helpful when discriminating between malignant and benign lesions (15).

The following four pathological features are used to grade scrotal smooth muscle tumors: i) size ≥5 cm in dimension; ii) infiltrating margin; iii) ≥5 mitotic figures per 10 high-power fields; and iv) moderate cytological atypia. Tumors with only one of the aforementioned features are considered benign; those fulfilling two of the criteria are diagnosed as atypical or bizarre leiomyomas; and tumors exhibiting three to four of these criteria are classified as leiomyosarcomas (9). Immunohistochemistry is important in determining the nature of spindle cells and conferring a final diagnosis.

In conclusion, to our knowledge, only a small number of cases of bizarre leiomyomas of the scrotum have been reported in the literature thus far. This case report highlights diagnostic and treatment issues associated with this rare tumor type. Histologically, the tumor behaves differently to conventional leiomyomas and leiomyosarcomas; therefore, definitive diagnosis is established by pathological evaluation. Correct diagnosis is important to avoid overdiagnosis and unnecessary clinical treatment.

## Figures and Tables

**Table I:** Bizarre leiomyomas of the scrotum reported in the literature.

Case	First author (ref.)	Year	Age (years)	Diameter (cm)	Clinical features	Position	Pathology
1	Nishiyama (4)	1987	46	6	Painless mass for 20 years	Left	Bizarre nuclei
2	De Rosa (10)	1996	49	NA	NA	NA	Bizarre nuclei
3	Slone (12)	1998	53	3	Painless mass for several years	Left	Bizarre nuclei
4	Slone (12)	1998	58	2	Painless mass for several years	Right	Bizarre nuclei
5	Slone (12)	1998	44	2	Painless mass for 4 years	Right	Bizarre nuclei
6	Rodruiguez-Parets (13)	1997	NA	NA	NA	NA	Bizarre nuclei
7	Fadare (5)	2003	69	3	Painless mass for 5 years	Anterior	Bizarre nuclei
8	Kim (3)	2003	65	1	Accidental discovery	Left	Bizarre nuclei
9	Sevilla (6)	2004	43	3.5	Accidental discovery	NA	Bizarre nuclei
10	Cabello (11)	2004	75	10.6	Accidental discovery	Right	Bizarre nuclei
11	Celia (7)	2005	52	1.7	Painful mass for 1 year	Right	Bizarre nuclei
12	Masood (8)	2008	59	8.5	Painless mass for 18 years	Right	Bizarre nuclei
13	Philip (14)	2008	65	3	Painless mass for 4 weeks	Right	Bizarre nuclei
14	Rao (9)	2012	64	4	Painless mass for 6 months	Anterior	Bizarre nuclei

NA, not available.
